# Role of preoperative neutrophil to lymphocyte ratio in prediction of recurrence, progression, and BCG failure in non-muscle invasive bladder cancer: a retrospective study

**DOI:** 10.11604/pamj.2023.44.145.38621

**Published:** 2023-03-27

**Authors:** Idriss Ziani, Ahmed Ibrahimi, Youssef Zaoui, Hachem EL Sayegh, Redouane Abouqal, Yassine Nouini, Amal Bouziane

**Affiliations:** 1 Department of Urological Surgery “A”, Ibn Sina University Hospital of Rabat, Rabat, Morocco,; 2 Faculty of Medicine of Rabat, Mohammed V University in Rabat, Rabat, Morocco,; 3 Acute Medical Unit, Ibn Sina University Hospital, Faculty of Medicine and Pharmacy, Rabat, Morocco,; 4 Laboratory of Biostatistics, Clinical Research and Epidemiology, Mohammed V University in Rabat, Rabat, Morocco,; 5 Department of Periodontology, Faculty of Dental Medicine, Mohammed V University in Rabat, Rabat, Morocco

**Keywords:** Non-muscle invasive bladder cancer, neutrophil/lymphocyte ratio, biomarker, BCG failure, immunotherapy

## Abstract

**Introduction:**

neutrophil/lymphocyte ratio (NLR), as a biomarker of the systemic inflammatory response, has been studied for diverse tumors. Our study aims to determine whether the NLR can be reliably used as a tool to predict disease course in patients diagnosed with primary non-muscle invasive bladder tumors (NMIBC).

**Methods:**

a retrospective study between 2009 to 2014 was conducted on 300 patients newly diagnosed with NMIBC at our institution. The cut-off value of NLR was set at 2.5. Survival curves were compared using the log-rank test. The association between recurrence, progression, and NLR was assessed univariate, and the prognostic significance of high NLR was assessed using multivariate analysis.

**Results:**

one hundred and seventy-five patients had an NLR <2.5 and 125 patients had an NLR ≥ 2.5. The survival rate with recurrence at 5 years was higher in the group with an NLR >2.5 (p<0.001, 35 vs 18 months), similarly, the survival rate with progression at 5 years was higher in the group with an NLR > 2.5 (p=0.001, 36 vs. 27 months). The failure rate of immunotherapy using BCG was higher when the NLR was over 2.5. In a multivariate analysis, the factors associated with recurrence were NLR>2.5 (HR=2.03, 95% CI=1.32-3.11, p=0.001), pathologic stage pT1 (HR=2.42, 95% CI=1.52-3.85, p=0.001), high-grade (HR=1.76, 95% CI=1.52-3.92, p=0.01), concomitant CIS lesions (HR=2.31, 95% CI=1.36-3.92, p=0.001), presence of lymphovascular emboli (HR=5.77, 95% CI=1.77-18.78, p=0.004), and BCG immunotherapy failure (HR=5.29, 95% CI=2.88-9.70, p=0.001). With regard to progression, in a multivariate study, the significant factors were NLR>2.5(HR=2.91, 95% CI=1.17-7.23, p=0.01), BCG immunotherapy failure (HR=5.68, 95% CI=3.16-10.22, p=0.001), and the presence of lymphovascular emboli (HR=5.01, 95% CI=1.50-16.05, p=0.001).

**Conclusion:**

preoperative NLR value could predict recurrence, progression, and failure of BCG immunotherapy in NMIBC patients.

## Introduction

Bladder cancer is the 7^th^ most frequent cancer diagnosed in men and the 11^th^ in both genders [1]. At diagnosis, 75% to 85% of bladder tumors are non-muscle invasive cancer (NMIBC) and the remaining 15% to 25% are invasive [2]. Transurethral resection of bladder tumor (TUR-BT) is the recommended treatment for NMIBC. A second resection called “second look” is usually done when indicated. Depending on the risk stratification, intravesical instillation of immunotherapy may be indicated according to the French Urology Association (AFU) and European Association of Urology (EAU) guidelines [3,4].

The dilemma in treating NMIBC is to preserve the bladder and its function while acknowledging the risk of recurrence (78% of cases) and progression to muscle-invasive disease (45% of cases) [5]. Identifying patients with a higher risk of recurrence or progression will help predict the outcome and adapt the treatment proposed aiming for the best response [5]. Many predictive factors influencing recurrence and/or progression have been identified by the literature such as the stage and the grade of the tumor(s), its size and number, and the existence of carcinoma in situ (CIS) lesions at diagnosis [6,7]. These factors alone cannot judge a more aggressive attitude; moreover, it does not make it possible to select patients potentially resistant to BCG therapy [7,8].

Numerous biomarkers of inflammation have been studied to predict the evolution and progression of the neoplastic disease, such as the neutrophil-lymphocyte ratio (NLR). In patients with solid tumors, a high ratio is strongly correlated with locally advanced disease and both a lower relative survival rate and disease-specific survival rate [9]. In regards to NMIBC, in the meta-analysis, a high NLR is associated with a higher rate of recurrence and progression, as well as the failure of adjuvant therapies [10]. However, its use as a biomarker in NMIBC still needs to be validated by larger studies. This paper aims to determine if a high preoperative NLR in NMIBC cases is a reliable biomarker in predicting the recurrence and progression of the neoplastic disease and to help forecast the response to intravesical instillation of Bacillus Calmette-Guérin (BCG) therapy.

## Methods

**Study design and setting:** this is a retrospective cross-sectional study including 300 patients who underwent transurethral bladder resection (TURBT) at Ibn Sina University Hospital, Rabat, Morocco over a period of 5 years, from January 2009 to December 2014, and was diagnosed histopathologically with a primary NMIBC. Additionally, all patients diagnosed with intermediate or high-risk NMIBC followed by intravesical BCG protocol consisting of induction treatment (6 weekly instillations) followed by monthly maintenance treatment for one year or three years. Depending on the high risk, postoperative follow-up consisted of cystoscopy and imaging of the upper urinary tract performed every 3 months the first 2 years, every 6 months 2 to 5 years after surgery, and annually thereafter.

**Participants:** all patients with a newly diagnosed NMIBC (Ta-1 stage) that meet the inclusion criteria and follow-up until December 2019 were enrolled.

**Exclusion criteria:** patients with a history of anemia, hematological malignancies, HIV seropositive or documented auto-immune disease, patients diagnosed either with an intermediate risk NMIBC and received Doxorubicin, patients with only carcinoma in situ (CIS) lesions at the anatomopathological study of the TURBT specimen, patients with follow-up were short <6 months after initial resection, and incomplete pathological information were excluded.

**Data collection:** clinicopathologic variables included age, gender, smoking history (categorized as former and current smokers compared with nonsmokers), and preoperative neutrophil/lymphocyte ratio from a complete blood count, were collected. The operative report registry was consulted to obtain data concerning the characteristics of the tumors such as the macroscopic aspect, tumors´ size (above or below 3cm), and number and location of the tumor(s). Tumors were graded according to the 1973 WHO grading system, staged according to the 2002 American Joint Committee on Cancer TNM staging system, and the presence of CIS lesions or vascular emboli [3,4].

**Risk-adapted management:** all the patients underwent a TURBT. A re-resection (second look) was done when indicated two to six weeks after the first resection. European Association of Urology (EAU) and French Association of Urology (AFU) risk stratification was used to determine if the patients would benefit from control cystoscopy only or would receive intravesical instillation of BCG (six installations then maintenance for 12 to 36 months) [3,4]. The risk scores for tumor recurrence and progression were calculated according to the European Organisation For Research And Treatment of Cancer (EORTC) risk tables, based on the number of tumors, tumor size, prior recurrence rate, T category, concurrent CIS, and tumor grade [11]. Patients with a progression risk score of 0, 2 to 6, and ≥7 were categorized as low, intermediate, and high risk for progression, respectively, according to the European Association of Urology guidelines [10]. Similarly, patients with a recurrence risk score of 0, 1 to 9, and ≥10 were categorized as low, intermediate, and high risk for recurrence, respectively [11].

**Study endpoints:** our endpoints were recurrence, progression, and failure of BCG therapy.

**Definitions:** Bacillus Calmette-Guérin (BCG) failure was defined according to AFU recommendations by any early high-grade recurrence (<12 months from TURBT), late recurrence (unless the second TURBT shows the absence of residual high-grade lesion), or progression to an invasive tumor. However, early or late low-grade recurrence was not considered BCG failure [4,5]. A disease recurrence was described as the first pathologically confirmed tumor relapse in the bladder, regardless of tumor stage. Disease progression was defined as a rise in the T category from CIS or Ta to T1 (lamina propria invasion), the development of ≥T2 or lymph node (N) disease or distant metastasis (M1), or an increase in grade from low to high [11].

**Statistical analysis:** the qualitative variables were expressed in number and percentage, and the quantitative variables were expressed in mean and standard deviation and/or median and interquartile range. The cut-off value of the NLR used in this study was 2.5 (≥2.5v s <2.5), as mentioned in most previous reports in the literature [10]. Clinical and pathological characteristics of patients were stratified by the NLR cutoff point. Categorical variables were compared using the Chi-square test or Fisher´s exact test when appropriate, and continuous variables were compared using the independent samples t-test. The survival analysis for recurrence, progression, and failure of BCG treatment between the two groups (NLR ≥ 2.5 vs. NLR < 2.5) were constructed by the Kaplan-Meier method and compared by the log-rank test. The prognostic varieties in predicting recurrence and progression were assessed by univariate analysis using the statistical log-rank test and multivariate analysis using Cox regression. All statistical tests were two-sided, and a significant difference was considered when P < 0.05. Statistical analysis of our data was performed by the statistical software “IBM SPSS Statistics V. 20.00.”

**Ethical approval:** our study obtained the favorable opinion of the Ethics Committee for Biomedical Research of the Faculty of Medicine and Pharmacy; the University of Rabat under the reference CERB 24-22 dated 03/22/2022. All patients provided informed written informed consent with guarantees of confidentiality.

## Results

**General characteristics of the study population:** of the 300 patients who were included in our study, 81.3% were males (n=244) and 18.7% were females (n=56). The mean age of patients was 58.95±9.87 years. The NLR was <2.5 in 58% of the patients (n=175) and ≥2.5 in 42% of the patients (n=125). The median NLR value was 2.20 (1; 5). 130 (43%) patients received BCG immunotherapy, and 48 of them (16%) presented with BCG failure later on, of the 48 patients who failed BCG treatment, 30 received induction and maintenance therapy, while 18 received only induction therapy and presented early recurrence before starting maintenance treatment. One hundred and fifty-eight patients (55.6%) presented a recurrence of their disease at a median time of 26 months (IQR: 6-37), and 44 (15%) had progression of their disease at a median time of 16 months (IQR: 9-40), and 31 patients underwent a cystoprostatectomy with a median follow-up in this group of 105 months. Table 1 presents the clinicopathologic characteristics of the patients included in the study according to the value of the NLR.

**Table 1:** clinicopathologic characteristics of cohort stratified by preoperative NLR

Neutrophil/lymphocyte ratio			
**Variable**	**≥2.5 N=125 (42.0)**	**<2.5 N=175 (58.0)**	**Total N=300 (%)**
**Age (years)**	58.20 (±9.66)	59.49 (±10.20)	58.95 (±9.87)
**Tobacco use**	160 (65.0)	85 (35.0)	245 (81.7)
**Sex**	170 (70.0)	74 (30.0)	244 (8.3)
Male			
Female	26 (46.0)	30 (54)	56 (19.0)
**Pathologic T stage**	80 (46.0)	94 (54.0)	174 (58.0)
pTa			
pT1	90 (72.0)	36 (28.0)	126 (42.0)
**Grade**	100 (76.0)	31(24.0)	131(43.0)
High grade			
low grade	72 (43.0)	97(57.0)	169 (56.0)
**BCG immunotherapy**	70(54.0)	60 (46)	130 (43.0)
**BCG failure**	30 (62.0)	18 (38)	48 (37.0)
**CIS lesions**	35 (80.0)	9 (20.0)	44(15.0)
**Focality**	98 (60.0)	56 (40)	154 (51.0)
Multiple			
Unique	38 (27.0)	100 (73.0)	138 (46.0)
**Tumor size**	93 (64.0)	52 (36.0)	145 (48.0)
>3cm			
<3cm	54 (35)	101(65)	155 (52.0)
**Cystoprostatectomy**	16 (50)	15 (50)	31(10.3)
**Recurrence**	102 (65)	56 (35)	158 (48.0)
**Progression**	30 (69)	14 (31)	44 (14.0)

NLR= Neutrophil/lymphocyte ratio; CIS=carcinoma in situ; pTa= papillary tumor confined to the epithelial layer of varying grade; pT1= papillary tumor invading the lamina propria of varying grade; BCG: Bacillus Calmette-Guérin

**Figure 1:**
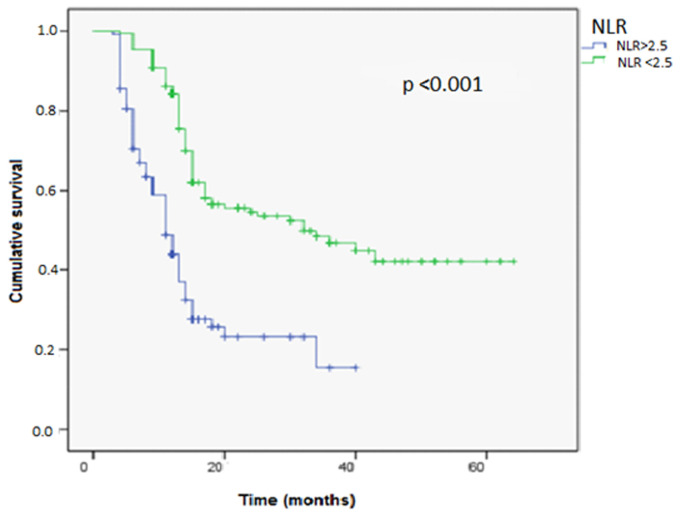
tumor recurrence-free survival curve in both groups NLR > 2.5 Vs NLR < 2.5 using the log-rank model

**Figure 2:**
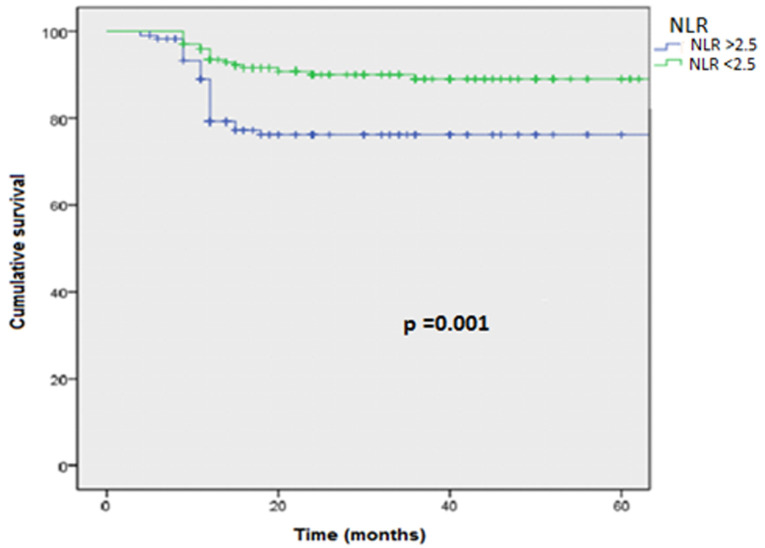
tumor progression-free survival curve in both groups NLR > 2.5 Vs NLR < 2.5 using the log-rank model

**Survival analyses:** the survival rate with recurrence at 5 years was higher in the group with an NLR ≥2.5, and the mean survival time without recurrence was 35 months in the group with an NLR <2.5 compared to 18 months in the group with an NLR≥2.5 and was statistically significant (p<0.001, 18 vs35 months) (Figure 1). Similarly, the survival rate with progression at 5 years was higher in the group with an NLR ≥2.5, with a mean survival time of 36 months compared to 27 months in the group with an NLR≥2.5 and was statistically significant (p=0.001; 27 vs 36 months) (Figure 2).

**Figure 3:**
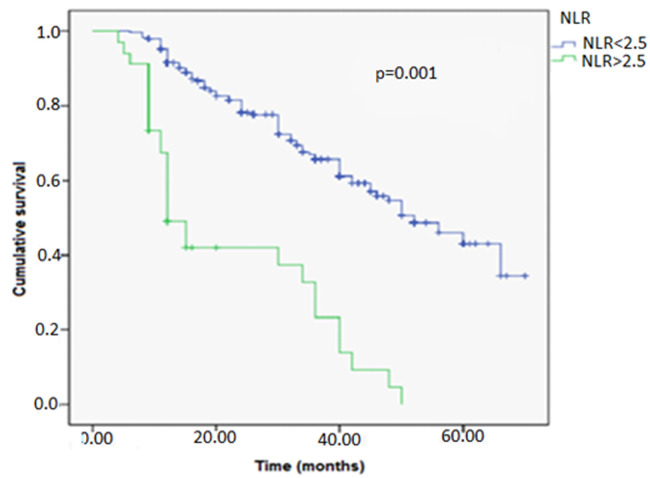
BCG failure-free survival curve in both groups NLR > 2.5 Vs NLR < 2.5 using log-rank model

In our study, the NLR influenced the response to BCG immunotherapy. Of the 130 patients who received intravesical instillations of BCG, 48 of them (16%) were resistant to it. In the group with an NLR <2.5, the mean survival time without BCG failure was 38 months, compared to 29 months in the group with an NLR ≥2.5 and was statistically significant (p=0.001; 29 Vs 38 months) (Figure 3). The mean survival time without failure was higher in the group with an NLR <2.5.

**Figure 4:**
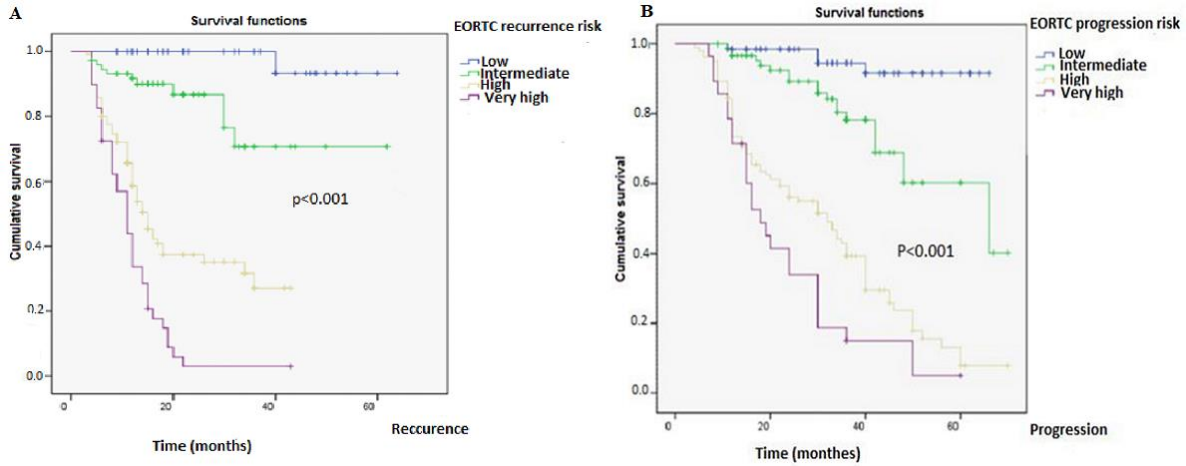
survival curves indicating the probability of recurrence; A) and progression; B) as a function of NLR according to the EORTC risk model

**European Organisation for Research and Treatment of Cancer risk stratification:** the risk stratification allowed us to identify an association between an NLR ≥2.5 and a high or very high risk of recurrence or progression of the disease. Among the patients (n=44) whose tumors´ risk stratification was very high, 96% (n=42) of them had an NLR ≥2.5. On the other hand, in the patients whose tumors risk stratification was low, 94% (n=51) had an NLR <2.5 (Table 2). A statistically significant association between the risk of recurrence and the risk of progression calculated using EORTC risk stratification and an NLR ≥2.5 was also found statistically significant (P<0.001) (Figure 4 A and B).

**Table 2:** recurrence and progression risk according to EORTC depending on the NLR

(EORTC risk)	Recurrence (%)		Progression (%)	
	NLR<2.5 (n=175)	NLR≥2.5 (n=125)	NLR<2.5 (n=175)	NLR≥2.5 (n=125)
**Low**	58 (33)	4 (3)	72(41)	4 (3)
**Intermediate**	76 (43)	20 (16)	70(40)	20 (16)
**High**	38 (22)	76 (60)	30(16)	76 (60)
**Very high**		25 (20)	3 (2)	25 (20)
**P-value**		<0.001		<0.001

EORTC= European Organisation For Research And Treatment of Cancer; NLR= Neutrophil/lymphocyte ratio

**Factors associated with tumor recurrence:** our analysis objective was to identify predictive factors for disease recurrence. An NLR ≥2.5 was found to be a reliable predictive factor for disease recurrence in both univariates (HR=2.59, 95% CI=1.81-3.70, p=0.001) and multivariate analysis (HR=2.03, 95% CI=1.32-3.11, p=0.001). In univariate analysis, predictive factors for disease recurrence were pathologic stage pT1 (HR=2.42, 95% CI=1.68-3.50, p=0.001), high-grade tumors (HR=1.46, 95% CI=1.02-2.09, p=0.03), multiple lesions (HR=1.73, 95% CI=1.17-2.56, p=0.006), tumor size >3cm (HR=1.46, 95% CI=1.03-2.07, p=0.03), concomitant CIS lesions (HR=2.82, 95% CI=1.82-4.26, p=0.001), presence of lymphovascular emboli (HR=13.56, 95% CI=4.43-41.49, p=0.001), and BCG immunotherapy failure (HR=6.61, 95% CI=4.12-10.62, p=0.001). In multivariate analysis, by adjusting the significant factors in univariate analysis (NLR>2.5, pathologic stage pT1, high grade, BCG failure, lymphovascular emboli, and CIS), all the factors aforementioned were found to be predictive of disease recurrence except a tumor size >3cm (p=0.37) and multiple lesions (p=0.07) (Table 3).

**Table 3:** predictive factors of recurrence in univariate and multivariate study using Cox's proportional-hazards regression model

Variable	Univariate analysis			Multivariate analysis		
	HR	95% CI	P-value	HR	95% CI	P-value
**NLR ≥2.5**	2.59	1.81-3.70	0.001	2.03	1.32-3.11	0.001
**Pathologic stage pT1**	2.42	1.68-3.50	0.001	2.42	1.52-3.85	0.001
**High grade**	1.46	1.02-2.09	0.03	1.76	1.52-1.84	0.01
**Multiple lesions**	1.73	1.17-2.56	0.006	1.52	0.95-2.43	0.07
**Size >3cm**	1.46	1.03-2.07	0.03	0.82	0.52-2.43	0.37
**BCG failure**	6.61	4.12-10.62	0.001	5.29	2.88-9.70	0.001
**Lympho-vascular emboli**	13.56	4.43-41.49	0.003	5.01	1.50-16.05	0.001
**CIS**	2.82	1.82-4.26	0.001	2.14	0.99-4.62	0.051

CI= confidence interval; NLR= Neutrophil/lymphocyte ratio; CIS=carcinoma in situ; pT1= papillary tumor invading the lamina propria of varying grade; BCG: Bacillus Calmette-Guérin

**Factors associated with tumor progression:** a high NLR above 2.5 was also found to be predictive of tumoral progression in univariate (HR=2.17, 95% CI=1.27- 4.49, P=0.001) and multivariate analysis (HR= 2.91, 95%CI=1.17-7.23, p=0.01). In univariate analysis, we also identified as predictive factors for disease progression: size >3cm (HR=1.60, 95%CI=1.54-1.74, p=0.001), BCG immunotherapy failure (HR=3.53, 95% CI=1.39-8.74, p=0.03), lymphovascular emboli (HR=1.85, 95% CI=1.23-2.77, p=0.003) and concomitant CIS lesions (HR=1.85, 95% CI=1.73-2.3, p=0.001). By adjusting the most significant factors in multivariate analysis, failure of BCG immunotherapy (HR=5.68, 95% CI=3.16-10.22, p=0.001) and lymphovascular emboli (HR=5.01, 95% CI=1.50-16.05, p=0.001) were identified as significant factors (Table 4).

**Table 4:** predictive factors of progression in univariate and multivariate using Cox's proportional-hazards regression model

Variable	Univariate analysis			Multivariate analysis		
	HR	95% CI	P-value	HR	95% CI	P-value
NLR ≥2.5	2.17	1.27-4,49	0.001	2.91	1.17-7.23	0.01
Pathologic stage pT1	1.14	0.50-2.63	0.74	-	-	-
High grade	1.19	0.64-2.22	0.57	-	-	-
Multiple lesions	1.22	0.75-1.88	0.3	-	-	-
Size >3cm	1.60	1.54-1.74	0.001	0.33	0.23-1.46	0.71
BCG failure	3.53	1.39-8.74	0.03	5.68	3.16-10.22	0.001
Lympho-vascular emboli	1.85	1.23-2.77	0.003	5.01	1.50-16.05	0.001
CIS	1.85	1.73-2.3	0.001	2.14	0.99-4.62	0.051

NLR= Neutrophil/lymphocyte ratio; CIS=carcinoma in situ; BCG: pT1= papillary tumor invading the lamina propria of varying grade; Bacillus Calmette-Guérin

## Discussion

This study showed the prognostic value of the high NLR > 2.5 in the unfavorable evolution of NMIBC. Recurrence-free and progression-free survival was better in the NLR<2.5 group, respectively (18 vs. 35 months) and (27 vs. 36 months). Similarly, an NLR >2.5 was statistically significantly associated with recurrence and progression in univariate and multivariate analysis. A high NLR is associated with a tumor at a high or very high risk of recurrence and progression according to the EORTC risk calculator. In addition, a high NLR was predictive of resistance to intravesical immunotherapy by BCG. Patients with high NLR have had BCG failures precociously. As it concerns the cut-off of NLR, recent studies recommended an NLR cut-off value of 3. But the method for selecting the NLR cut-off value remained unclear [12]. Currently, there is no recommended cut-off used in current practice [13]. The average cut-off used in the studies was 2.5 and was similar to, Mbeutcha *et al*. study [14].

Regarding literature data, a metanalysis of 2298 NMIBC patients mentioned that a high preoperative NLR was associated with lower survival rates without recurrence in four of the studies (HR ¼ 2.31, 95% CI: 1.27-4.22, P< 0.006), and in two of them with lower survival rates without progression even in patients who received intravesical immunotherapy (HR=2.54, 95% CI: 1.36-4.71, P=0.003). This meta-analysis pointed out that NLR was associated with an increased risk of disease recurrence and progression in patients who underwent TURBT for NMIBC. Furthermore, NLR was an independent predictor of disease recurrence and progression in NMIBC treated with BCG patients. NLR could be used to improve clinical decision-making regarding treatment and follow-up scheduling [10]. Another study of 271 patients, mentioned that NLR value was opposed to anatomopathological pT1 stage and high grade and found that patients with an NLR >1.8 had a 1.5 times greater risk for lamina propria infiltrating tumors [15]. In a European retrospective multicentric study, including 918 patients, the prognosis value of NLR was evaluated in patients diagnosed with primitive NMIBC. The cut-off value used was 3. In univariate and multivariate analysis, an NLR ≥3 was significantly associated with a lower survival rate without recurrence or progression. A High NLR was also correlated with BCG immunotherapy failure. The authors of the paper proposed a predictive model that includes the NLR to help predict the risk of recurrence or progression of NMIBC, although they did exclude patients with CIS lesions [16].

In a prospective cohort study of 178 patients led by Favilla *et al*. the NLR was evaluated as a prognostic biomarker for follow-up of NMIBC, revealing a statistically significant association between an NLR ≥3 and the recurrence of the NMIBC but not for progression [13]. Ozyalvacli *et al*. reported in their 166 patients cohort study a statistically significant correlation between the recurrence of NMIBC pT1 and an NLR ≥ 2.43. Multivariate logistic regression analysis identified high NLR, multifocality of the lesions, and tobacco use as significant independent factors of recurrence. However, no statistically significant correlation between high NLR and progression was described [17]. Mbeutcha *et al*. demonstrated the existence of a link between immunotherapy response in NMIBC and systemic inflammatory biomarkers, including NLR. Authors evaluated retrospectively in a cohort of 1117 patients, the high recurrence and progression risk of NMIBC in patients with an elevated NLR. The association was confirmed in a subgroup of 300 patients who benefited from BCG intravesical instillations. They recommend adjoining the NLR to other prognostic markers [14]. Our results seem to match what is reported by the literature. An elevated NLR ≥2.5 was significantly associated with high to very high-risk bladder cancer and with lower survival rates without progression or recurrence.

As for the impact of the NLR on the response to BCG immunotherapy, the Vartolomei *et al*. meta-analysis corroborated the results of four studies that all linked an elevated NLR to an inferior response to BCG immunotherapy [10]. A retrospective study of 100 patients diagnosed with NMIBC and treated with trans-urethral resection followed by intravesical BCG instillations, demonstrated the prognostic value of NLR before the immunotherapy. The mean NLR in the group with a favorable response to immunotherapy was 2.61 ± 0.77 compared to 3.65 ± 1.16 in the group who didn´t respond to the treatment. The NLR was correlated to recurrence (r=0.55, p=0.01) and to progression risk scores (r=0.49, p=0.01) [18]. Compared to previously published studies, and in an aim to get meaningful results on the impact of an elevated NLR in the response to adjuvant BCG immunotherapy following TURB, we only included patients who underwent similar protocols of BCG intravesical instillations and excluded patients who received other adjuvant treatments such as chemotherapy. We also excluded patients who had a history of diseases that could affect the NLR. Our results seem to match previous reports.

In addition to the statistically significant association between an elevated NLR and high to high-risk tumors, our study demonstrated a statistically significant correlation between an NLR ≥ 2.5 with unfavorable prognostic factors such as CIS and lymphovascular emboli. Our results are in agreement with those already published in the literature thus supporting the prognostic value of a high neutrophil/lymphocyte ratio in the prediction of recurrence, progression, and failure of BCG immunotherapy in NMIBC [8,10,17,18]. These results reflect the role of lymphocytes and inflammation in the immune response to BCG and the process of carcinogenesis. The inflammatory response plays a key role in cancer initiation and development [19].

Cells involved in inflammation in particular, neutrophils and lymphocytes play a central role in the process of carcinogenesis and tumor development via a very complex interaction [20]. As in other studies, in our study, the neutrophil/lymphocyte ratio as a biomarker of inflammation proved its effectiveness as a prognostic biomarker of NMIBC [20,21]. Faced with these encouraging results, the question arises, are these conclusions found in the literature linked to a state of neutrophilia or rather to lymphocytopenia? This represents the main limitation of this NLR report. it would be wiser to conduct two separate studies, one studying neutrophilia as a biomarker and the other lymphocytopenia to identify each biomarker and its implication in the evolution of NMIBC.

**Limitations:** the retrospective nature constitutes the main limitation of our study. The small number of samples, non-randomized, and single referral institutions also represent limitations. Further prospective, well-controlled clinical studies of diverse patients in multiple institutions are required to validate the role of NLR as a prognostic marker, which may improve current risk stratification tools and treatment outcomes in this group of patients.

## Conclusion

Our study demonstrated that an elevated NLR is a predictive factor of the recurrence and progression of NMIBC. Furthermore, the NLR could identify patients who didn’t respond to BCG immunotherapy. All this makes an NLR a valuable biomarker that is simple to obtain, reproducible, and cost-effective. Still, our results need to be confirmed by larger multicentric prospective studies.

### What is known about this topic

- *Bladder cancer is the 7^th^ most frequent cancer diagnosed in men and the 11^th^ in both genders;*
- *Despite the different therapies available, recurrence and progression rates are still high reaching respectively 70% and 30%;*
- *The impact of biological markers of inflammation, in particular the NLR, is still poorly understood*.

### What this study adds

- *Our cohort study demonstrated that an elevated NLR is a predictive factor of the recurrence and progression of NMIBC;*
- *The NLR could identify patients who didn't respond to BCG immunotherapy*.
